# Influence of Magnetic Microparticles Isolation on Adenine Homonucleotides Structure

**DOI:** 10.3390/ma7031455

**Published:** 2014-02-25

**Authors:** Monika Kremplova, Dana Fialova, Lukas Nejdl, David Hynek, Libuse Trnkova, Jaromir Hubalek, Rene Kizek, Vojtech Adam

**Affiliations:** 1 Department of Chemistry and Biochemistry, Mendel University in Brno, Zemedelska 1, Brno CZ-613 00, Czech Republic; E-Mails: mkremplova@volny.cz (M.K.); dana.dospivova@seznam.cz (D.F.); lukasnejdl@gmail.com (L.N.); d.hynek@email.cz (D.H.); libuse@chemi.muni.cz (L.T.); hubalek@feec.vutbr.cz (J.H.); kizek@sci.muni.cz (R.K.); 2 Central European Institute of Technology, Brno University of Technology, Technicka 3058/10, Brno CZ-616 00, Czech Republic; 3 Department of Chemistry, Faculty of Science, Masaryk University, Kamenice 5, Brno CZ-625 00, Czech Republic

**Keywords:** adenine, adenine interaction, magnetic beads, square wave voltammetry, aptamer, biosensor, nanobiotechnology

## Abstract

The electroactivity of purine and pyrimidine bases is the most important property of nucleic acids that is very useful for determining oligonucleotides using square wave voltammetry. This study was focused on the electrochemical behavior of adenine-containing oligonucleotides before and after their isolation using paramagnetic particles. Two peaks were detected—peak A related to the reduction of adenine base and another peak B involved in the interactions between individual adenine strands and contributes to the formation of various spatial structures. The influence of the number of adenine bases in the strand in the isolation process using paramagnetic particles was investigated too.

## 1. Introduction

It is well known that a DNA molecule is composed of nucleotides, the basic building blocks of DNA. The nucleotide consists of 2-deoxy-β-d-ribose, phosphate group and purine (adenine, guanine) or pyrimidine (cytosine, thymine) base [1]. For the study of nucleic acids, various instrumental methods such as ultraviolet-visible (UV/Vis) spectrometry, electrophoretic methods, polymerase chain reaction (PCR) and circular dichroism are used [2–6]. Besides these methods, electrochemical methods are also possible to use [7]. Electroactivity of nucleic acids bases on mercury electrodes is one of the most sensitive ones. Palecek was the first who used modern oscillographic polarography for successful detection of redox DNA signals [8–11]. Since then, great progress and development has been made in the electrochemistry of nucleic acids on various electrodes [7,12,13]. The attention is paid to various electrochemical methods using a mercury electrode as a working one including linear sweep and cyclic polarography/voltammetry (elimination polarography/voltammetry), differential pulse polarography/voltammetry, square wave polarography/voltammetry, alternating currents (AC) polarography/voltammetry, and chronopotentiometry for analysis of DNA [7,14]. The connection of adsorptive transfer stripping technique with the above-mentioned methods is very promising tool for studying nucleic acids [14].

Square wave voltammetry (SWV) is one of the most sensitive electrochemical methods for determination of oligonucleotides (ODNs) [15–17]. SWV is generally the best choice among all pulse methods, because it offers background suppression combined with the effectiveness of differential pulse voltammetry (DPV), slightly greater sensitivity than that of DPV, much faster scan times, and applicability to a wider range of electrode materials and systems. The most reproducible behavior and lowest detection limits are generally found on mercury surfaces [18]. From the point of view of DNA electroanalysis, this method belongs to the most sensitive label free ones with the lowest limits of detection [19–24].

Aptamers represent one of the specific parts of the whole wide oligonucleotide group. They are defined as molecules of ribonucleic (RNA) and single-strand (ss) deoxyribonucleic (ssDNA) acids or peptides that can bind to targets with high affinity and specificity due to their specific three-dimensional structure [25]. Especially RNA and ssDNA aptamers can differ from each other in the sequence and the folding pattern, although they bind to the same target [26]. Applications of aptamers in the area of biosensing have been widely developed during last decade. Aptamers have been studied as a biomaterial in numerous investigations concerning their use as diagnostic and therapeutic tools, biosensing probe, and in the development of new drugs, mainly drug delivery systems [25,27,28].

A phenol-chloroform extraction is considered a standard method for nucleic acid isolation, but this method needs special laboratory equipment, and it is also time-consuming. For these reasons, the research has been focused on other alternative methods for biomolecules isolation [29–31]. One possible way is the isolation of nucleic acids using paramagnetic and/or superparamagnetic particles (MPs) [32,33]. Paramagnetic particles are the particles with size ranges from nm to mm, responding to an external magnetic field and facilitating the binding of bioactive molecules due to their modified surface by biocomponents [34–36]. The main advantages of MPs are as follows: easy to use, fast sample preparation without centrifugation and dialysis. Physicochemical properties of MPs are very important for their biological applications. The most commonly used MPs in biosensor applications are composed of ferrous oxide or ferric oxide [37]. Nanoparticles of ferric oxide can provide adequate surface for binding biomolecules. There are two methods of surface modifications of MPs. The first method is based on the electric envelope layer, which ensures the electrostatic adsorption of biomolecules [38]. The second method of surface treatment of paramagnetic particles is based upon biomolecules anchored on the particle, which are able to bind the target biomolecule specifically [23,39]. The isolation of adenine containing nucleotides is based on this principle, because oligo (deoxythymine) 25 is anchored on the surface of MPs and can be hybridized by molecules of adenine-containing nucleotides [12].

The aim of our study was to investigate the electrochemical behavior of adenine-containing nucleotides on the surface of a mercury electrode and their behavior after separation using paramagnetic particles. The description of this phenomenon could be useful for understanding of some aspects of isolation processes using magnetic materials.

## 2. Results and Discussion

### 2.1. Square Wave Voltammetry of Adenine-Containing Oligonucleotides

**Figure 1.**
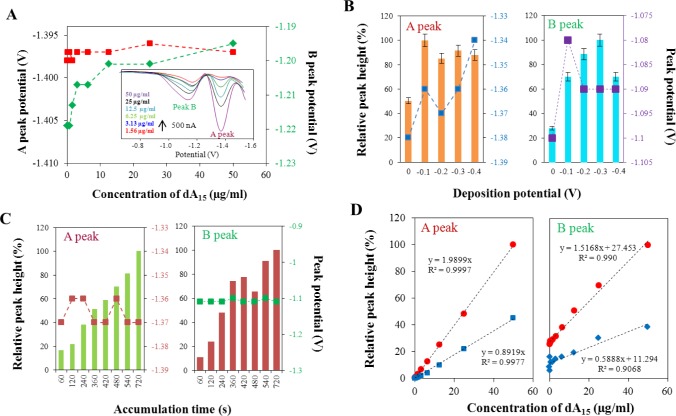
(**A**) Dependence of position of peak A and B of adenine-containing nucleotide dA15. Red points indicate potential values of peak A, green points indicate potential values of peak B, determined by square wave voltammetry with following parameters: start potential −0.1 V, end potential −1.6 V, deposition potential −0.1 V, accumulation time 720 s, equilibration time 5 s, voltage step 0.006 V, amplitude 0.02 V, frequency 280 Hz (sweep rate 1.6663 V/s). In inset: typical SW voltammograms of adenine-containing nucleotide dA15 at various concentrations; (**B**) Dependence of relative height and potential of peak A and B on the applied deposition potential for dA15 at concentration 25 μg/mL; (**C**) Dependence of a relative height and potential of peak A and B on the accumulation time for dA15 at concentration 25 μg/mL; (**D**) Influence of applied frequency on intensity of peak A and B for dA15 at concentration 25 μg/mL. Two different frequencies were applied, 100 (blue points) and 280 Hz (red points).

As it was mentioned above, square wave voltammetry is one of the most frequently used and sensitive electrochemical methods for determination of DNA [22,40,41]. For oligonucleotides, containing different bases, both CA and G peaks can be measured using this method. Current peaks of guanine can be obtained on mercury or amalgam electrodes by re-oxidation of the product of guanine reduction, 7,8-dihydroguanine [42]. Inset of Figure 1A shows typical voltammograms of oligonucleotide that contains adenine bases. Homonucleotide dA_15_ was selected as an example. Two peaks were recorded: the first one at potential −1.37 V, well-known and often described as peak A (related to adenine reduction on HMDE) and the second one recorded at potential −1.10 V, described as peak B in this study. In Figure 1A, it is also shown that the potentials of both the peaks are measured within the concentration range from 0.09 to 50 μg/mL of dA_15_. For peak A, there are no changes in the peak potential with the increasing concentration of the adenine oligonucleotide dA_15_. On the contrary, the potentials of peak B are shifted to more positive values with increasing concentration of dA_15_. The absolute potential difference, determined in the concentration range from 0.09 to 50 μg/mL of dA_15_, was 0.025 V.

Palecek defined and described the peak II_SW_, which has high similarity (in accordance with the obtained voltammograms) with our peak B, only in a native double-stranded DNA structure [24,43]. In our samples, only adenine single-stranded oligonucleotides were occurring. Due to this fact, we assumed the detection of the peak B as a result of formation of more complex structures of the adenine chains. The interactions in the nucleosides structures, in general, can be considered as electrostatic interactions. These interactions lead to the formation of planar base-pair structures as those of the Watson-Crick (WC) type [44]. One of them, the formation of the *trans*-configuration of adenine-adenine pairs, is expected, when the phosphodiester bonds have the same orientation. In addition, the non-covalent interactions that stabilize both DNA and RNA can be divided into three groups as follows: hydrogen bonding, base-stacking, and electrostatic effect of the strands [44]. The base-stacking interaction is also recognized as crucial to stabilize the structure of nucleic acids. It is particularly important to note that the planar structures are generally more stable than stacked structures. These effects have been shown in numerous studies of behavior of the adenine bases and there are several possibilities of formation of complexes [45–51], mainly possible hydrogen bonds in A–A structures [52,53].

According to the angle between the planes of two bases, five structural types of pairing of bases can be distinguished: (1) planar, H-bonded; (2) non-planar, H-bonded; (3) T-shaped; (4) planar stacked; and (5) non-planar stacked [54]. The planar base-pair structure for dimers of bare adenine has been observed and assigned to that having two moieties that are doubly hydrogen bonded [53]. *Plutzer et al.* [53] identified various conformations of adenine pair by IR-UV resonance spectroscopy. Some of the isomers are stabilized by the inter-fragment interactions similar to the H-bonding. Five of them have hydrogens of –NH_2_ of the one fragment pointing towards nitrogen atoms of the other fragment. However, the study of structures revealed that these bonds are relatively long and, consequently, are weaker than regular hydrogen bonds [51]. A symmetric H-bonded dimer, labelled as AA-HB1, is the most stable adenine dimer [51,52,54]. The stacked structures, stabilized dimers of adenine nucleosides, have been widely investigated and related to the various energy changes by some authors [50,51]. The results demonstrate that the dimer possesses a stacked structure being stabilized by the formation of hydrogen-bonding network involving the two sugar groups. In many presented variations of adenine and thymine dimers, the adenine dimer AA-ST1 was suggested as the most stable stacked configuration [51].

#### Dependence of Measured Signals on Different SWV Parameters

All measurements were carried out using square wave voltammetry. To use the most sensitive conditions for analysis of the compounds of interest, we optimized some parameters. Deposition potential was the first optimized parameter (Figure 1B). Five different values of potential, at which the effect of sample deposition on the mercury drop on the electrochemical signal was investigated, were selected. The highest and best A signal was detected using the deposition potential of −0.1 V. This potential was used in all further measurements. Other selected deposition potentials showed lower signals or potential shift at different concentrations of the sample occurred. On the other hand, the peak B had the best signal at the potential of −0.3 V. The difference between both the best accumulation potentials indicates the different nature of both signals. Because of the fact that the peak A has been well described and known, we decided to realize next measurements with the accumulation potential of −0.1 V. The second factor in support of this decision was that the SWV scans were performed from 0 to −1.6 V and in such case the accumulation at −0.1 V causes no problems, but only lower signal.

The effect of accumulation time of monomeric adenine deoxyribonucleotides on the surface of mercury electrode was the second studied parameter. Figure 1C clearly shows the increasing trend of the electrochemical signal depending on the increasing time of the accumulation. The tested time interval was from 60 to 720 s. The accumulation time of 720 s provided the highest signal and therefore was chosen as the best time of accumulation. The potentials of the signals during the varying accumulation time showed no significant changes.

The frequency is a very important parameter in determination of DNA by the square wave voltammetry. Figure 1D shows the effect of frequency on the size and quality of both A and B peaks. Two values of frequency, 100 and 280 Hz, were used in this experiment. The intensity of signal was measured within the concentration range from dA_15_ 0.09 to 50 μg/mL. The electrochemical signal at the frequency of 280 Hz was two times higher in comparison to that measured at the frequency of 100 Hz for both the peaks. Therefore, the frequency 280 Hz was used for all further measurements.

### 2.2. Influence of Number of Adenine Bases on the Electrochemical Signal

**Figure 2.**
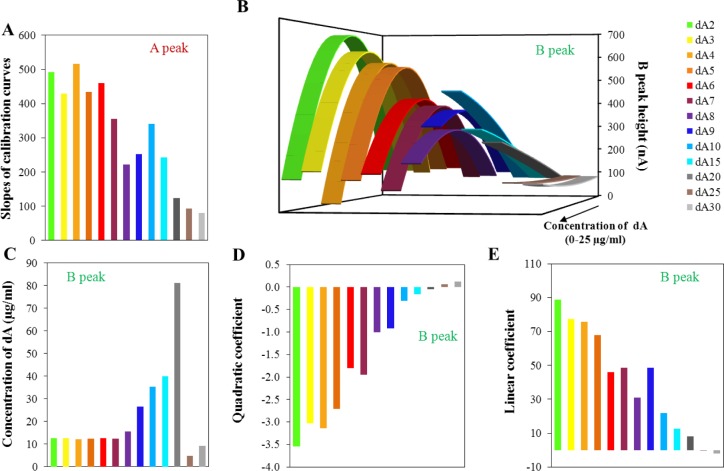
(**A**) Slopes of linear concentration dependencies obtained from evaluation of intensity of peak A within the concentration range from 50 ng/mL to 25 μg/mL of adenine nucleotide; (**B**) Calculated quadratic dependencies based on the measured data obtained for the peak B within the concentration range from 50 ng/mL to 25 μg/mL of adenine nucleotide; (**C**) Determined concentrations of adenine nucleotides related to the local maxima/minima of calculated quadratic dependencies for peak B; not restricted to model concentration interval of adenine nucleotide; (**D**) Dependence of quadratic coefficient calculated from quadratic dependencies on various number of adenine bases in strand; (**E**) Dependence of linear coefficient of calculated quadratic dependencies on the various number of adenine bases in strand.

Thirteen oligonucleotides differing in the number of adenine bases in their sequence were used in this study, oligonucleotides dA_2_–dA_10_, then dA_15_, dA_20_, dA_25_, and dA_30_. For each ODN, calibration curves were determined within the concentration range from 50 ng/mL to 25 μg/mL. The applied amount of ODN, quantitatively determined using UV/Vis spectrometry [20,55–57], (data is not shown) served as a control. The influence of ODN concentration on peak A and B height was determined. The reduction signal of adenine (peak A) changed linearly with increasing ODN concentration and peak B had quadratic concentration dependences. The slopes of the calibration curves related to peak A depending on the length of the oligonucleotide chain are shown in Figure 2A. From oligonucleotide dA_2_ to dA_4_, we can assume that the slope of the calibration curve of peak A have similar values with a slightly declining trend. Generally, the slopes of the calibration curves gradually decreases with the increasing number of adenine bases in the oligonucleotide chain, thus the sensitivity of the method for this determination is limited.

Calibration curves were also evaluated for the peak B. Figure 2B shows the calculated quadratic calibration curves. The quadratic regression equations were based on the calibration set of data of the individual oligonucleotides with confidence interval at least 0.99. Generally, the increasing length of the adenine oligonucleotide strand causes reduction of the maximum electrochemical signal and its shift to the lower intensity. Nevertheless, for the set of dA_2_–dA_7_ the shift of the maximum is not relevant and only intensity of the signal decreases. This phenomenon could be explained by the modification of the surface of the electrode by adenine nucleotides. The longer strands (dA_7_–dA_30_) cause decrease of the intensity of the signal of peak B, which indicates “isolation” character of longer strands towards forming bonds between individual adenine strands. For short the adenine strands, there is a prerequisite for the pairing of adenine bases between two strands. The electron transport through the strands is a complex process and the transmission onto the surface of the electrode is not fully understood. For the longer adenine strands, there is apparently no connection between adenine bases in two strands, but the adenine-adenine connection can be formed in one strand. From dA_8_ there is a probability of the intra-molecular binding of adenines due to the conformational freedom in individual adenine strands and thus the electrochemical signal of peak B is lowered. Peak B, as it has been assumed and confirmed by Palecek’s experiments [24,43], is connected with the formation of binding between various strands. The shape of the obtained curves changed dramatically for the borderline between dA_20_ and longer oligonucleotides. The successive lengths of the oligonucleotide strand (dA_25_ and dA_30_) present only minimal changes in the intensity of the peak B with the increasing concentration of dA. This effect could be caused by the forming of relatively stable spatial intra-conformation of individual strands that is stabilized by A–A bonds. The formation of inter-strand binding is, in this case, unlikely and unexpected due to high conformational freedom in the individual adenine strands. Such structures could easily cover the surface of the electrode and thus eliminate the response to the concentration changes.

For the model of quadratic equations, the concentrations related to the local maxima/minima were calculated (Figure 2C). This calculated concentration is necessary to understand as an optimal amount of individual dA for the most creation of space structures based on adenine interactions. As it is obvious from the figure, dA_2_–dA_7_ does not change the calculated concentration maxima. It means that the same concentration of dA (variable in the length of chain) induces the maximum of peak B measured (variable in the peak intensity), it means maximal spatial arrangement of dA molecules. From eight adenines in the strand (dA_8_), the concentration maxima increase rapidly up to twenty adenines, where the breakpoint of the shape of the calculated curves is present. Further, the local minimum is presented for dA_25_ and dA_30_. The length of 20 adenines is critical in the obtained curve shape too. As it is indicated in Figure 2B, the shape of the curve is changed with the increasing number of adenines in the strand. The length of twenty adenines is the borderline between the negative and positive quadratic parts of the calculated curves (Figure 2D) and thus limits the shape of the obtained curves. The linear parts of the calculated dependencies (Figure 2B) are shown in Figure 2E. These dependencies directly reflect the changes in the length of the strand.

As it was mentioned above, the shape of the calculated curves changes with the increasing number of adenines. The change between dA_20_ and dA_25_ is obvious from Figure 2C–E. Among these two values, one curve lies having zero quadratic part of the calculated equation (linear equation). This represents the break point of modeling curves. The calculation of this point was based on the direct connection of points related to the dA_20_ and dA_25_ in the dependence of quadratic part on the number of adenines in the strand (Figure 2D). This equation is as follows: *y* = 0.0215*x* − 0.4809. From this equation, it was simply calculated that the length of 22 (exactly 22.37) adenine bases is critical for the formation of the inter-stranded structure of the homoadenine strands. This conclusion is based on the belief in the suggested explanation of the observed changes in the intensity of the signal of peak B, which is described above. Similarly, it is possible to calculate the critical number of adenines from the dependence of the linear part on the number of adenines in the strand (Figure 2E). This equation is as follows: *y* = −1.7512*x* + 43.2220. The critical number of adenines of 25 was calculated from this equation. This result is probably influenced by errors in a greater extent, because the linear part of the model quadratic equations still remains at the critical point (change from quadratic to the linear equation). Therefore, the obtained result seems to be correct. This conclusion is supported just with a look to Figure 2C, where the change is located between dA_20_ and dA_25_.

**Figure 3.**
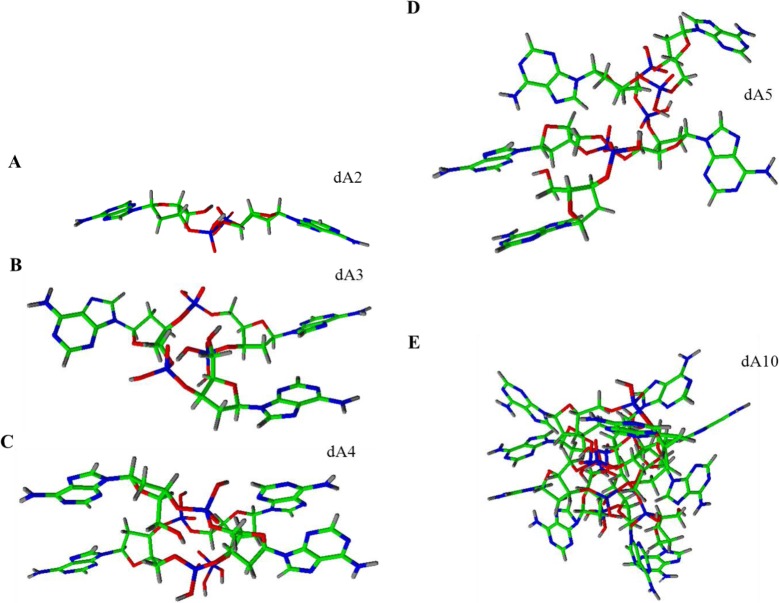
Modelling of various dA structures created with software ACD/ChemSketch using functions as follows: clean structure, check of tautomeric form and 3D optimization. Model of 3D structures for various lengths of (**A**) dA_2_; (**B**) dA_3_; (**C**) dA_4_; (**D**) dA_5_ and (**E**) dA_10_. Individual elements are represented by various colours as follows: hydrogen (black), nitrogen (blue), carbon (green), phosphorus (red).

For the better understanding and imagination of conformation of the strands, we attempted to create structure models of individual homoadenine strands (Figure 3) in software ACD/ChemSketch using functions as follows: clean structure, check of tautomeric form, and 3D optimization. From the presented models, it is obvious that the adenine bases are located to the surface of creating structure. The dA_2_ creates the planar structure. The other homoadenines create spherical structures, which are obvious with the steps from dA_2_ (Figure 3A) to dA_5_ (Figure 3D), and next to the dA_10_ (Figure 3E). The dA_10_ structure is spherical at most from the presented models only. The higher dA structures are more complex and the spherical shape is confirmed (not shown).

### 2.3. Isolation of Adenine-Containing Oligonucleotides

**Figure 4.**
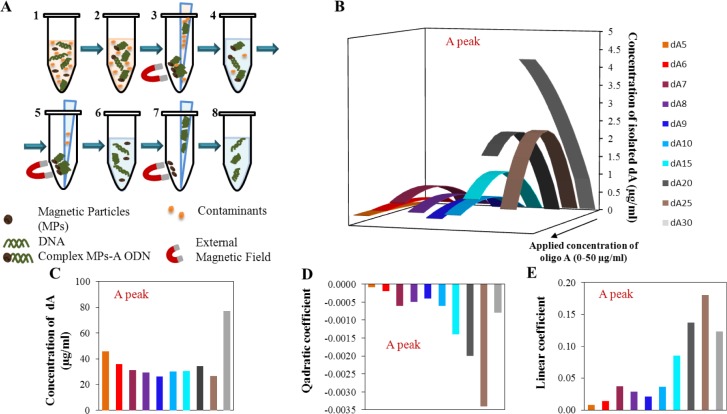
(**A**) Scheme of procedure of isolation using magnetic particles (MPs)—**1** mixture of adenine nucleotides with MPs, **2** immobilization of adenine nucleotides to MPs, **3** removing contaminants, **4** washing MPs coupled adenine nucleotide, **5** removing contaminants, **6** elution of adenine oligonucleotides from MPs, **7** removing adenine oligonucleotides, **8** purification; (**B**) Dependence of concentration of isolated adenine nucleotides related to the applied concentration of adenine nucleotides. The calculated quadratic dependences are based on the data measured for peak A within the concentration range from 50 ng/mL to 25 μg/mL of adenine nucleotides before isolation process; (**C**) Determined concentration of adenine nucleotides related to the local maxima/minima of calculated dependences for peak A after isolation process; not restricted to model concentration interval of adenine nucleotides; (**D**) Dependence of quadratic coefficient of calculated quadratic dependences on various number of adenine bases in strand (related to part B); (**E**) Dependence of linear coefficient of calculated quadratic dependences on various number of adenine bases in strand (related to part B).

The second part of our experiment was focused on the isolation of adenine ODNs using paramagnetic particles (scheme is shown in Figure 4A). The application of paramagnetic particles is widespread in the biotechnology procedures and experiments. This is mainly due to the simplicity of the procedure, low costs, and good efficiency. Our aim was to investigate the behavior of the adenine strand after the isolation process, mainly with focus on both the monitored peaks. The model procedure of the isolation process was adapted from Huska *et al.* [58]. With regard to the expected lower isolation yield, concentration range 1.56–50 μg/mL of all mentioned ODNs was used.

#### 2.3.1. A Peak

In Figure 4B, calculated quadratic dependence of peak A after isolation by MPs is recorded. The first important result is that no signal for the oligonucleotides dA_2_, dA_3_, and dA_4_ was detected after isolation, so the magnetic separation cannot be done for these short ODNs. In comparison with the calibration dependencies of peak A for the oligonucleotides without isolation, the calibration curves after isolation changed course from pseudo-linear (very few quadratic) to quadratic with the increasing number of adenines and the electrochemical signal reached higher values for the longer oligonucleotide strands. This is the second important result as the change from linear calibration dependence before isolation to quadratic ones after the isolation process. This phenomenon is probably caused by the binding of individual adenine strands onto the dT_25_ located on the surface of MPs. It seems that the longer dA strands have higher affinity due to the higher number of established A–T bonds. The quadratic course of the dependences is probably caused by the saturation of the surface of magnetic particles. The reduction of the electrochemical signal at higher concentrations is apparently the result of the supersaturation of the surface where the ODNs are tore off out from the magnetic particles.

The maximum yield of the individual dA after isolation (Figure 4C) was observed within the concentration range from 27.3 μg/mL to 47.1 μg/mL of applied dA concentration. An exception is the oligonucleotide dA_30_, where the maximum yield was calculated at the applied concentration 77.7 μg/mL; this concentration is beyond the applied concentration range, so this value represents only the theoretical calculation based on the quadratic equation of the calibration curve for the respective oligonucleotide (Figure 4C). In Figure 4D,E, the individual parameters of evaluated quadratic curves are demonstrated. The obvious change of the quadratic part is located between the dA_25_ and dA_30_, which is in accordance with the calculated concentration of the maximum signal (Figure 4C). Linear parts of the quadratic equations respect the same phenomenon and the break point is obvious for the dA_30_ too (Figure 4E).

#### 2.3.2. B Peak

**Figure 5.**
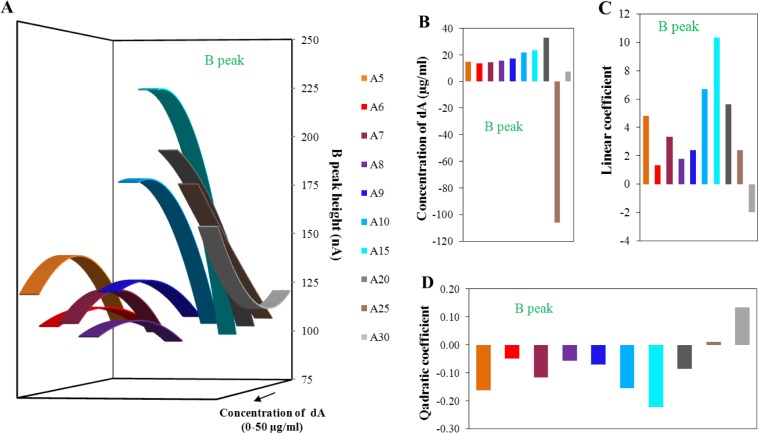
(A) Dependence of intensity of peak B related to the applied concentration of adenine nucleotides. The calculated quadratic dependencies are based on the measured data for peak B within the concentration range from 50 ng/mL to 25 μg/mL of adenine nucleotides before isolation process; (**B**) Determined concentration of adenine nucleotides relate to the local maxima/minima of calculated dependencies for peak B after isolation process; not restricted to model concentration interval of adenine nucleotides; (**C**) Dependence of linear coefficient of calculated quadratic dependencies on various number of adenine bases in strand (related to part B); (**D**) Dependence of quadratic coefficient of calculated quadratic dependencies on various number of adenine bases in strand (related to part B).

The evaluation of peak B is shown in Figure 5 in the same way as for peak A in Figure 4. The obtained quadratic curves for various lengths of adenine strands were related to the various concentrations of the dA (Figure 5A). It is obvious that the presented curves change their shape with the increasing number of adenines in the strand. The shapes of individual dA curves presented in Figure 5A are similar to the stage before the isolation. While the intensity of the peak decreased before the isolation process with the number of adenines in the strand, after isolation the peak intensity growth. This change could be connected with the dominance of the magnetic beads isolation effect. It means the effect of probe length, located on the surface of magnetic beads which better binds longer dA. However, the change of intensity of peak B for individual monomeric adenine deoxyribonucleotides depending on concentration is lower in comparison to the stage before the isolation. This effect is connected with the magnetic beads isolation efficiency which decreases the obtained heights of peak B.

Dependence of the dA concentration related to the local maxima/minima on the number of adenines in strands is presented in Figure 5B. Here, the optimal concentrations of individual dA slightly increased with increasing number of adenines in strands. The great change in obtained concentration for dA_25_ was caused by the great proximity of this strand length to the break-point of this dependence. This borderline, important change of the obtained curves, was determined to be 24 adenine bases. This number was calculated from the elimination of quadratic coefficient (zero value of the coefficient) from modeling quadratic curves. The theoretical number of adenines calculated from the linear coefficients was determined as 29 adenines. These calculated borderlines correspond approximately to the state before the isolation. This difference is greater than for the determination without the isolation process. The differences among the number of adenines determined from the quadratic or linear part of the function before and after the isolation process is two adenine bases. Such variation is able to relate the isolation procedure itself and not to the change of dA strands behaviors. It is important to note that these borderlines could denote the number of adenines in strand, which change the character of adenines interaction form intermolecular to intramolecular. In comparison to the results obtained before the isolation, dependencies of the individual equation parts (quadratic and linear—Figure 5C,D) after the isolation are more incorrect and less tendentious. The dispersion of individual values is obvious in the interval from dA_5_ to dA_9_. From this reason, it could be assumed that the isolation process using MPs influences mainly the structure of adenine nucleotides in the range from the dA_5_ to dA_9_. From the dependencies presented in Figure 5A, it seems to be that dA_10_ and dA_15_ had the best conformation properties itself and for the binding of the dT_25_ probes, located on the surface of magnetic beads. In conclusion, the isolation process using paramagnetic particles is able to slightly influence and change the secondary structure of adenine homonucleotides. This fact can be very helpful in future studies of aptamer structures from the viewpoint of electrochemical detection.

### 2.4. Quantification of Isolation Procedure

**Figure 6.**
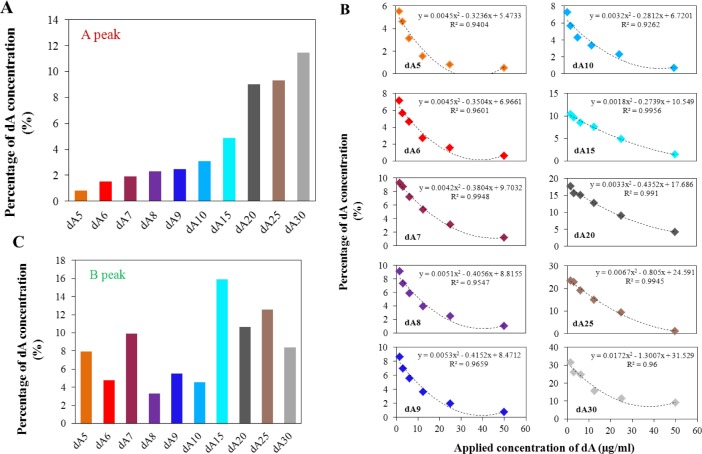
(**A**) Percentage of concentration of adenine nucleotides after isolation process (related to the applied concentration) depends on the number of adenine bases in strand—evaluation based on peak A; applied concentration of adenine nucleotide was 25 μg/mL; (**B**) Percentage of adenine nucleotides concentration after isolation process (related to the applied concentration) depends on applied concentration of adenine oligonucleotides—evaluation based on peak A; (**C**) Percentage of concentration of adenine nucleotides after isolation process (related to the applied concentration) depends on number of adenine bases in strand—evaluation based on peak B; applied concentration of adenine nucleotide was 25 μg/mL.

The process of the isolation is closely connected to the question of the efficiency of the isolation. We chose the initial concentration 25 μg/mL for all adenine ODNs to evaluate the efficiency of the isolation. The effect of the number of adenines in the strand was investigated in accordance with peak A and B (Figure 6A,B).

From the electrochemical signals, obtained after the isolation, the percentage of isolated amount was calculated. As it is well evident from Figure 6A, the efficiency of the isolation process increased with increasing length of the oligonucleotide strand. The highest efficiency of the isolation was demonstrated for the oligonucleotide dA_30_, the value at applied concentration was about 12%.

Figure 6B shows the percentage yield of the isolation for all applied concentrations of ODNs. For all adenine oligonucleotides, the dependence of percentage yield on the applied concentration shows polynomial character, confidence intervals varied from 0.92 to 0.99. The increasing applied concentration led to a decrease of the percentage yield. In contrast, the highest efficiency of the isolation process is well evident for the application of lower concentrations of adenine nucleotides. This is probably caused by the good steric access of the lower number of dA to dT strands located on the surface of magnetic particles. The longer dA strands had the greater affinity to the immobilized dT strands due to the higher number of A–T pairs.

After the separation of the various adenine nucleotides using the MPs, the electrochemical signal related to peak B was also evaluated. Figure 6C shows the dependence of intensity of peak B after isolation process on the length of the adenine strands. The presented data shows no significant correlation between the intensity of peak B and the number of adenines in the strand. This experiment showed that peak B, in contrast to peak A, is not suitable for quantification of the adenine strands before/after the isolation process.

## 3. Experimental Section

### 3.1. Chemicals

All the chemicals in ACS purity were purchased from Sigma-Aldrich Chemical Corp. (St. Louis, MA, USA) unless noted otherwise. The deionized water was prepared using reverse osmosis equipment Aqual 25 (Brno, Czech Republic). The deionized water was further purified by an apparatus MiliQ Direct QUV (Aqua Osmotic, Tisnov, Czech Republic) equipped with an UV lamp. MiliQ water was applied for the dilution of samples. Synthetic homo ODNs in the range from 5’-AA-3’ (dA_2_) to 5’-A…A-3’ (dA_30_) were used as a standard. Stock standard solutions of ODNs (100 μg/mL) were prepared from lyophilized dA (0.5 mg/mL) with water of ACS purity (Sigma-Aldrich, St. Louis, MA, USA) and stored in dark at −20°C. The concentration of dA was determined spectrophotometrically at 260 nm using a spectrometer Spectronic Unicam (Spectronic Camspec Ltd, Leeds, UK). The pH values were measured using WTWinoLab Level 3 with terminal Level 3 (Wissenschaftlich-Technische Werkstatten, Weilheim, Germany).

### 3.2. Instrumentation for Isolation of Adenine-Containing Oligonucleotides (dA_2_–dA_30_)

The isolation of adenine-containing oligonucleotides was carried out using paramagnetic particles Dynabeads Oligo (dT)25 (Invitrogen, Carlsbad, CA, USA) and a magnetic stand Dynal Magnetic Particle Concentrator-S supplied by Dynal A.S (Oslo, Norway). All the experiments with paramagnetic particles were performed in a RNA/DNA UV cleaner box UVT-S-AR (Biosan, Riga, Latvia). For centrifuging and vortexing of a sample, a multi-spin MSC- 3000 centrifuge (Biosan, Riga, Latvia) placed in the UV cleaner box was used. Denaturation was carried out at 85 °C using Thermomixer 5355 Comfort/Compact (Eppendorf, Hamburg, Germany). The buffers used in our experiments were as follows: (a) phosphate buffer I: 0.1 M NaCl + 0.05 M Na_2_HPO_4_ + 0.05 M NaH_2_PO_4_; (b) phosphate buffer II: 0.2 M NaCl + 0.1 M Na_2_HPO_4_ + 0.1 M NaH_2_PO_4_; (c) acetate buffer: 0.2 M CH_3_COOH + 0.2M CH_3_COONa. Hybridization solution: 100 mM Na_2_HPO_4_ + 100 mM NaH_2_PO_4_, 0.5 M NaCl, 0.6 M guanidinium thiocyanate, 0.15 M Trizma base adjusted by HCl to pH 7.5.

### 3.3. Fully Automated Isolation of Adenine Contain Oligonucleotides (dA_2_–dA_30_)

A fully automated isolation was carried out on an automated pipetting system epMotion 5075 (Eppendorf, Hamburg, Germany). The position of B4 is a magnetic separator (Promega, Mannheim, Germany). The positions of C1 and C4 can be thermostated (Epthermoadapter PCR96, Eppendorf, Hamburg, Germany). The pipetting provides a robotic arm with adapters (TS50, TS300 and TS1000, Eppendorf, Hamburg, Germany) and Gripper (TG-T, Eppendorf, Hamburg, Germany). The samples are placed in the position B3 in an adapter Ep0.5/1.5/2 mL. A Module Reservoir is located in the position B1, where washing of solutions and waste are available. An ep*Motion* control panel controls the device. Tips are located in the A4 (ePtips 50), A3 (ePtips 300), and A2 (ePtips 1000) positions. PCR 96 plates are used. The resulting volumes of the collected samples ranged from 10 to 30 μL depending on the procedure.

### 3.4. Electrochemical Determination of Peak A

The determination of adenine-containing oligonucleotides was performed using a 797 VA Stand instrument connected to an 889 IC Sample Center (Metrohm, Herisau, Switzerland). An analyzer (797 VA Computrace from Metrohm, Herisau, Switzerland) employs a conventional three-electrode configuration with a hanging mercury drop electrode (HMDE) as a working electrode with drop area of 0.4 mm^2^, Ag/AgCl/3MKCl as a reference electrode, and a platinum auxiliary electrode. Metrohm supplies the following setup assembled of automated voltammetric analysis. A sample changer (Metrohm 889 IC Sample Center, Metrohm, Herisau, Switzerland) performs the sequential analysis of 96 samples in plastic test tubes. For the addition of standard solutions and reagents, an automatic dispenser (Metrohm 800 Dosimat, Metrohm, Herisau, Switzerland) is used, when a peristaltic pump station (Metrohm 843 Pump Station, Metrohm, Herisau, Switzerland) is employed for transferring the rinsing solution into the voltammetric cell and for removing solutions from the voltammetric cell. A central unit (Metrohm 846 Dosing Interface, Metrohm, Herisau, Switzerland) controls automatic dispenser and peristaltic pump station.

Square wave voltammetric measurements were carried out under the following parameters: deoxygenating with argon 120 s; start potential −0.1 V; end potential −1.6 V; deposition potential −0.1 V; accumulation time 720 s; amplitude 0.02 V; voltage step 5.951 mV; equilibration time 5 s; frequency 280 Hz (sweep rate 1.6663 V/s), volume of injected sample 20 μL; cell was filled with 1500 μL of electrolyte [0.2 M acetate buffer (CH_3_COONa + CH_3_COOH) adjust to pH 5].

### 3.5. Mathematical Treatment of Data and Estimation of Detection Limits

The mathematical analysis of the data and their graphical interpretation were analyzed by software Matlab (version 7.11., MathWorks, Natick, MA, USA). The results are expressed as mean ± standard deviation (S.D.) unless noted otherwise (EXCEL^®^, Microsoft, Redmond, WA, USA). The limits of detection (3 signal/noise, S/N) were calculated, whereas N was expressed as standard deviation of noise determined in the signal domain unless stated otherwise [59].

## 4. Conclusions

Isolation of nucleic acids or oligonucleotides via magnetic particles is one of many applications of these magnetic materials. In this study, the electrochemical behavior of adenine homonucleotides before and after the magnetic separation was described. The application of SWV method gave two different characteristic signals in the obtained voltammograms. The separation process based on paramagnetic particles influenced the behavior of adenine homonucleotides in the way of changes in structure (interruption of inter- and intra-stranded binds) and changes in analytical parameters (calibration curves). It seems that the separation processes based on magnetic materials could change the properties of biomolecules and influence especially their usage in *in vivo* applications.
